# Disrupted Functional Brain Connectivity in Partial Epilepsy: A Resting-State fMRI Study

**DOI:** 10.1371/journal.pone.0028196

**Published:** 2012-01-05

**Authors:** Cheng Luo, Chuan Qiu, Zhiwei Guo, Jiajia Fang, Qifu Li, Xu Lei, Yang Xia, Yongxiu Lai, Qiyong Gong, Dong Zhou, Dezhong Yao

**Affiliations:** 1 Key Laboratory for NeuroInformation of Ministry of Education, School of Life Science and Technology, University of Electronic Science and Technology of China, Chengdu, China; 2 Department of Neurology, West China Hospital of Sichuan University, Chengdu, China; 3 Department of Neurology, The Affiliated Hospital of Hainan Medical College, Haikou, China; 4 Key Laboratory of Cognition and Personality, Ministry of Education, School of Psychology, Southwest University, Chongqing, China; 5 Department of Radiology, Huaxi MR Research Center (HMRRC), West China Hospital of Sichuan University, Chengdu, China; Indiana University, United States of America

## Abstract

Examining the spontaneous activity to understand the neural mechanism of brain disorder is a focus in recent resting-state fMRI. In the current study, to investigate the alteration of brain functional connectivity in partial epilepsy in a systematical way, two levels of analyses (functional connectivity analysis within resting state networks (RSNs) and functional network connectivity (FNC) analysis) were carried out on resting-state fMRI data acquired from the 30 participants including 14 healthy controls(HC) and 16 partial epilepsy patients. According to the etiology, all patients are subdivided into temporal lobe epilepsy group (TLE, included 7 patients) and mixed partial epilepsy group (MPE, 9 patients). Using group independent component analysis, eight RSNs were identified, and selected to evaluate functional connectivity and FNC between groups. Compared with the controls, decreased functional connectivity within all RSNs was found in both TLE and MPE. However, dissociating patterns were observed within the 8 RSNs between two patient groups, i.e, compared with TLE, we found decreased functional connectivity in 5 RSNs increased functional connectivity in 1 RSN, and no difference in the other 2 RSNs in MPE. Furthermore, the hierarchical disconnections of FNC was found in two patient groups, in which the intra-system connections were preserved for all three subsystems while the lost connections were confined to intersystem connections in patients with partial epilepsy. These findings may suggest that decreased resting state functional connectivity and disconnection of FNC are two remarkable characteristics of partial epilepsy. The selective impairment of FNC implicated that it is unsuitable to understand the partial epilepsy only from global or local perspective. We presumed that studying epilepsy in the multi-perspective based on RSNs may be a valuable means to assess the functional changes corresponding to specific RSN and may contribute to the understanding of the neuro-pathophysiological mechanism of epilepsy.

## Introduction

Epilepsy is a brain disorder characterized predominantly by recurrent and unpredictable interruption of normal function, and affects a variety of mental and physical functions [1]-[3]. There are many different types of epileptic seizures, and a patient may suffer one or several of them. The intention of seizures’ classification implicates etiology, approaches to diagnostic evaluation, treatment and prognosis. The division, which epileptic seizures fall into two broad categories: generalized seizures (GS) and partial seizures (PS) [4] depends on which part of the brain is affected by initial activation, was widely used in clinic. Scalp electroencephalogram (EEG) was used in the context of epileptic disorder soon after it was discovered. But its low spatial resolution precludes the acquisition of detailed localization information. Functional magnetic resonance imaging (fMRI) which measures local hemodynamical changes, provide a powerful technique to localize the brain regions during an experimental condition noninvasively. Applications of fMRI to epilepsy have received considerable attention [5]–[7]. Especially, the combining fMRI with EEG was widely used to detect abnormal epileptic activations in the brain [8]–[10].

The spontaneous low frequency BOLD fluctuation, derived from resting-state fMRI data has received increasing interest [11]. In contrast to task-driven approach, the resting-state scans is relatively simple and easy to execute, and it is important for patients with cognitive dysfunction or physical impairment, who was not capable of performing tasks accurately [12], [13]. The spontaneous neuronal interaction were first investigated in motor cortices[14] and then extended to other cortical systems comprising visual and auditory networks, default mode network(DMN), attention and memory related regions [11], [13], [15]–[19]. These spatially segregated brain regions that exhibit spontaneous low frequency fluctuations were defined as ‘resting-state networks’ (RSN), and it implicated that the RSN may represent some underlying or intrinsic forms of brain functional connectivity in discrete neuroanatomical systems [11]. Up to now, it has been suggested that at least 10 to 12 RSNs can be detected from the brain cortex in resting-state fMRI. As a popular data analysis method of resting-state fMRI, functional connectivity evaluating temporal correlations between spatially discrete brain regions has been studied increasingly in healthy individuals [11], [14]–[16] as well as in patients with brain disorders [20]–[24].

In previous studies focused on epilepsy, the pioneer work reported the disturbance of language network in temporal lobe epilepsy (TLE) [7]. Subsequently, many works have implicated abnormalities in perceptual networks (visual, auditory and sensorimotor network) [25], DMN [26]–[28] and dorsal attention network (DAN) [29] in TLE. Additionally, in our previous studies, we found the abnormalities of DMN and basal ganglia network in patients with idiopathic generalized epilepsy [30], [31]. However, these studies were all focused on single functional network, such as DMN, perceptual network, few study investigated the interaction between the RSNs in epilepsy, which was possible means to understand globally the neuro-pathophysiological mechanism of epilepsy. Recently, the dysfunctional connectivity among multiple brain regions is considered as a central feature, and the International League Against Epilepsy Commission on Classification and Terminology uses the terminology ’distributed networks’ to descript the epilepsy [32]. The abnormal functional connectivity within RSNs or between them may be a feature of epilepsy from resting-state fMRI.

An extension of functional connectivity, called functional network connectivity (FNC), was developed [33]. FNC is powerful to characterize distributed changes in the brain by examining the interactions among different RSNs. Jafri and his colleagues conducted FNC analysis in schizophrenia, and found significant differences between patients and controls reflecting deficiencies in cortical processing in patients [33]. In the present study, 16 partial epilepsy patients were recruited to explore the functional connectivity within RSNs and between. RSNs were isolated using group ICA; then functional connectivity and FNC analysis were conducted in TLE group (7 patients) and mixed partial epilepsy group (MPE, 9 patients) to address the following questions: What is the influence of the TLE imposed on the functional connectivity of each RSNs and the FNC between RSNs? Is the influences identified in 1) existed universally in various partial epilepsy?

## Results

### Identifications of the RSNs for HC and TLE

**Figure 1:**
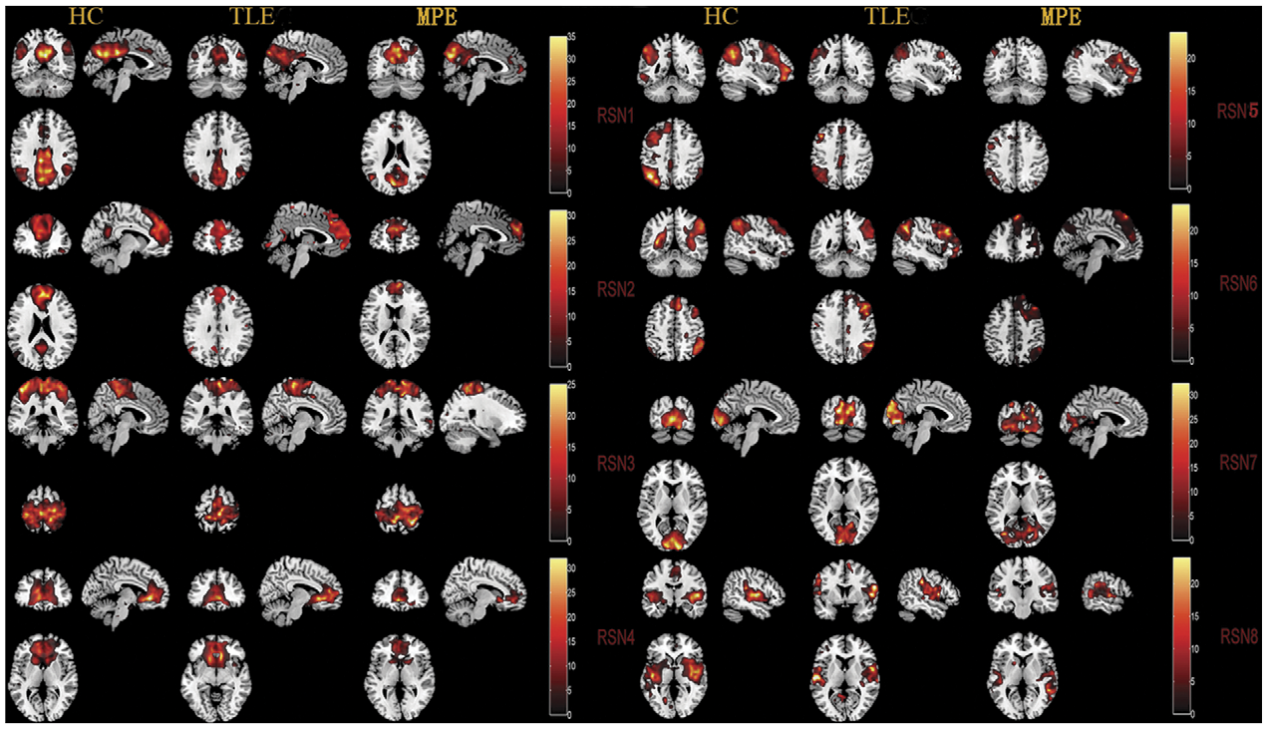
One-sample t-tests results of the eight RSNs in three groups (HC,TLE and MPE). Activated foci are shown with a significance threshold set at P<0.05 (corrected by FDR). The identified networks are labeled as follows: (RSN1) posterior part of the DMN, (RSN2) anterior part of the DMN, (RSN3) sensorimotor network, (RSN4) self-referential network, (RSN5) left lateral frontoparietal network, (RSN6) right lateral frontoparietal network, (RSN7) visual network, (RSN8) auditory network. The left side of the image corresponded to the left side of the brain.

After removing components which showed obviously artifactual patterns or ventricle regions, eight components (RSN1–RSN8) were selected for further analyses. The spatial maps of the eight RSNs are illustrated in Figure 1 for three groups (HC, TLE and MPE). The identified networks were labeled as follows.

RSN1: the posterior part of the DMN involved the posterior cingulate cortex (PCC), bilateral inferior parietal gyrus, angular gyrus;

RSN2: the anterior part of the DMN included primary clusters in the superior frontal gyrus and medial frontal gyrus. Typically, as an important network, DMN mainly encompasses PCC, the anterior cingulate cortex (ACC), and the bilateral inferior parietal lobule (IPL). It is interesting that the DMN was splitted into 2 components in current study, the anterior areas (RSN1) and the posterior areas (RSN2). A similar decomposition of the DMN has been observed previously [17], [34]–[36].

RSN3: sensorimotor network was a network corresponding to sensory-motor function [14], [37]. This network includes pre- and postcentral gyrus, the primary sensorimotor cortices, and the supplementary motor area.

RSN4: self-referential network putatively related to self-referential mental activity mainly including the medial-ventral prefrontal cortex, the pregenual anterior cingulate [38].

RSN5: left lateral frontoparietal network along with right lateral frontoparietal network showed the similar spatial patterns with DAN consisting of regions previously known to be involved in goal-directed top-down processing [39], [40]. This network primarily involved precuneus, inferior parietal lobule, middle frontal gyrus, superior parietal lobule.

RSN6: right lateral frontoparietal network including clusters lateralized to the right hemisphere putatively associated with DAN. Left lateral frontoparietal network and right lateral frontoparietal network were the only maps to be strongly lateralized, and were largely left–right mirrors of each other.

RSN7: visual network showed spatial patterns consisting of the middle temporal, superior temporal, insular and postcentral cortex which were previously known to be involved in visual processing.

RSN8: auditory network primarily encompassed middle temporal gyrus, superior temporal gyrus, insular and temporal pole, and corresponded to the auditory system.

### Group comparisons of functional connectivity within RSNs

**Figure 2:**
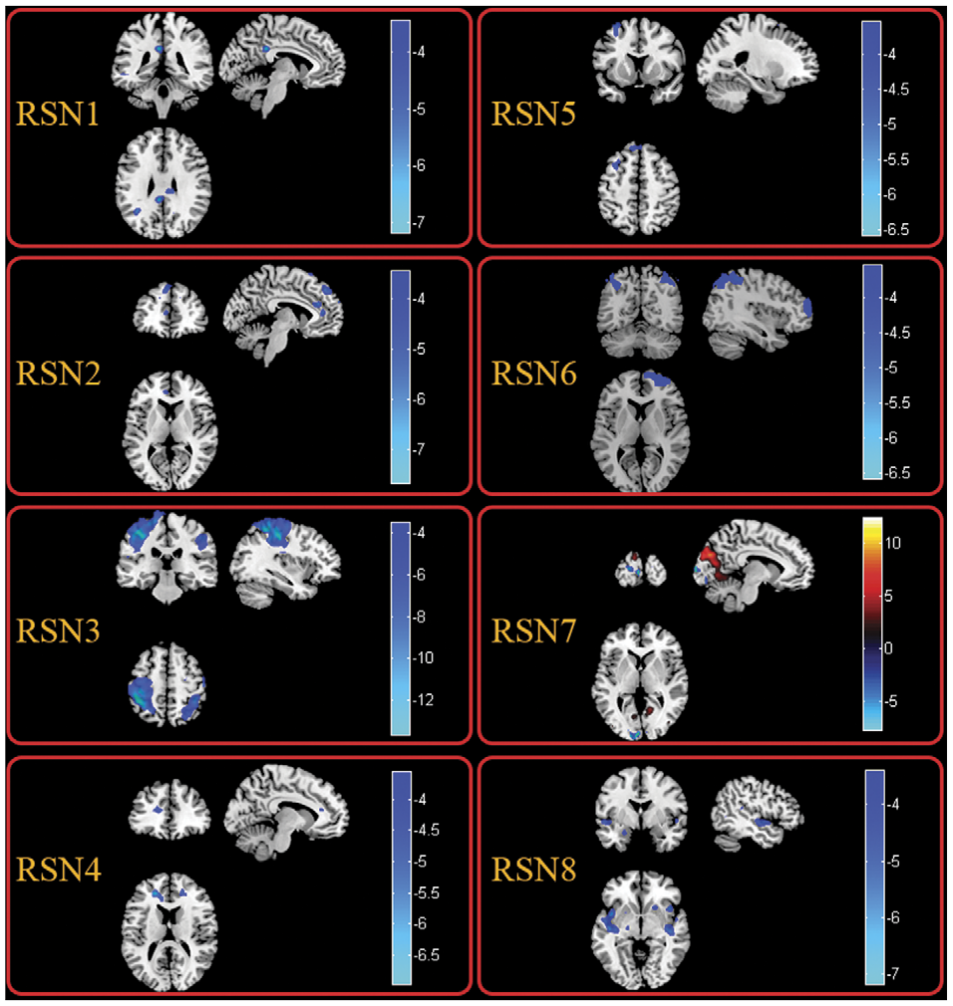
Two-sample t-tests results of the eight RSNs between TLE and HC (P<0.05 corrected by FDR). The warm and cold colors indicate the brain regions with significantly increased and decreased functional connectivity in TLE compared with HC, respectively. The left side of the image corresponds to the left side of the brain.

Between-group analysis of RSNs was performed using two-sample t-test. Figure 2 revealed the difference between TLE and HC in all the eight RSNs. We found that all the networks revealed decreased functional connectivity within the regions of each network in TLE. Among the eight RSNs, the visual network was the network not only revealing reduced functional connectivity but also increased functional connectivity in lingual gyrus, cuneus in bilateral occipital lobe. Table 1 summarized the significantly difference functional connectivity regions of each RSN with the Brodmann areas in which activations occurred, peak location and peak t value. Although previous studies have discussed the differences of functional connectivity between patients with temporal lobe epilepsy and healthy controls in DMN, perceptual and dorsal attention networks [25], [29], this work is the first to examine the functional connectivity alteration in, self-referential network and the frontoparietal networks which has been presumed to be related to attention function in patients with TLE.

**Table 1:** Significant difference of functional connectivity of the eight RSNs between HC and TLEG.

RSNs	Patients with TLE vs Controls			
	Brain regions	Brodmann	Peak locationMNI (x , y, z)	Peak t value
RSN1	Left Posterior Cingulate	26	−2, −41,28	7.16
	Left Angular Gyrus	39	−39, −54,33	5.54
	Left Superior Parietal Lobule	7	−35, −55,48	5.45
RSN2	Left Superior Frontal Gyrus	6	0,48,48	7.51
	Left Anterior Cingulate	11,24	−6,36,24	5.82
	Right Superior Frontal Gyrus	6	6,15,72	5.70
	Left Superior Frontal Gyrus	6,8	−3,24,59	4.37
RSN3	Left Postcentral Gyrus	3	−45, −36,60	13.56
	Left Superior Parietal Lobule	2	−21, −44,63	10.54
	Right Postcentral Gyrus	1,3	51, −18,57	8.56
	Left Precentral Gyrus	6	−34, −22,63	8.42
	Left Inferior Parietal Lobule	40	−35, −48,54	8.13
	Right Inferior Parietal Lobule	40	35, −39,51	7.19
	Right Superior Parietal Lobule	7	24, −59,57	5.23
RSN4	Left Superior Frontal Gyrus	9	−21,42,18	6.87
	Right Superior Frontal Gyrus	9	18,45,18	5.07
RSN5	Left Middle Frontal Gyrus	6	−30,12,66	6.51
	Left Superior Frontal Gyrus	8	−11,31,55	4.35
RSN6	Right Superior Frontal Gyrus	9,10	21,42,15	6.67
	Right Inferior Parietal Lobule	40	47, −48,52	5.74
	Left Inferior Parietal Lobule	39	−51, −63,39	4.94
RSN7	Left superior Occipital Gyrus	17	−8, −99,9	8.12
	Left Calcarine	17	−1, −98,9	5.63
	Left Lingual Gyrus*	17	−3, −90,0	11.95
	Left Cuneus*	18	−9, −102,9	10.39
	Right Lingual Gyrus*	18	15, −84, −12	8.54
RSN8	Left Insula	13	−42,6, −3	7.14
	Right Inferior Frontal Gyrus	47	42,15, −6	7.08
	Right Insula	13	42, −18,0	6.37
	Left Superior Temporal Gyrus	38	−24,3, −21	5.85
	Right Lentiform Nucleus	34	21,18, −3	5.44
	Left Subcallosal Gyrus	40	−24,9, −9	4.45
	Left Postcentral Gyrus	22	−50, −24,15	4.59

Note: * regions represented increased functional connectivity in TLE in contrast to HC.

**Figure 3:**
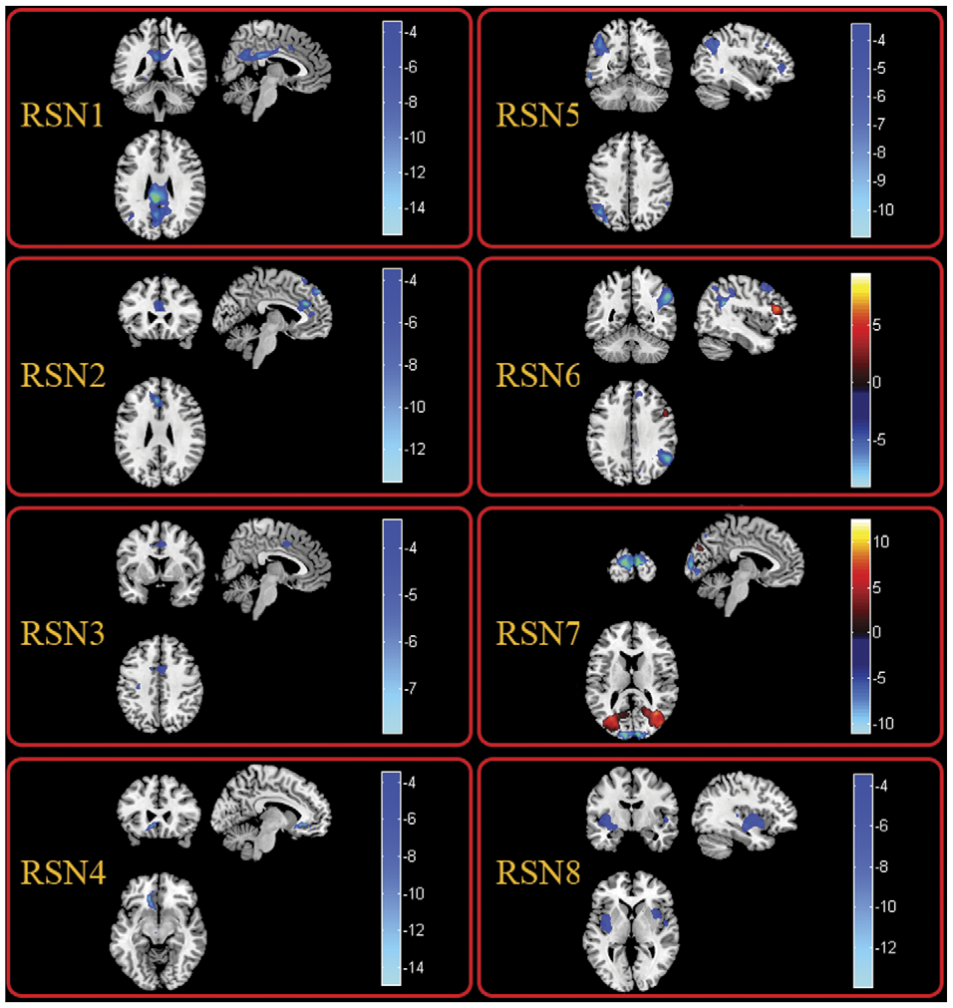
Two-sample t-tests results of the eight RSNs between MPE and HC (P<0.05 corrected by FDR). The warm and cold colors indicate the brain regions with significantly increased and decreased functional connectivity in MPE compared with HC, respectively. The left side of the image corresponds to the left side of the brain.

**Figure 4:**
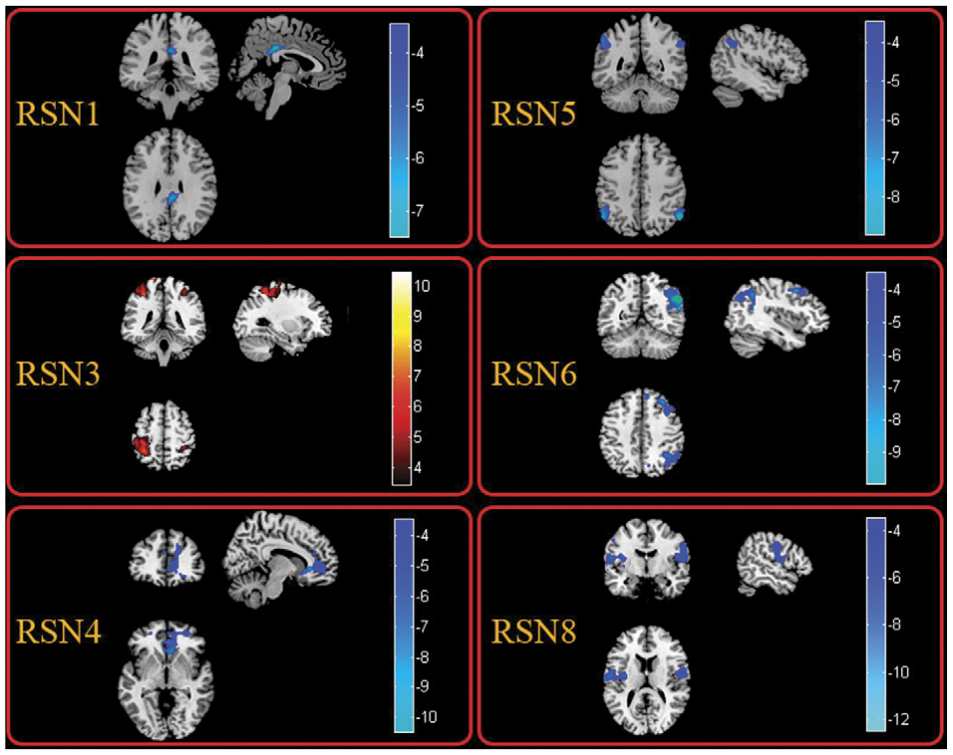
Two-sample t-tests results of the eight RSNs between MPE and TLE (P<0.05 corrected by FDR). The warm and cold colors indicate the brain regions with significantly increased and decreased functional connectivity in MPE compared with TLE, respectively. The left side of the image corresponds to the left side of the brain.

In order to examine whether the influences identified in TLE universally existed in the partial epilepsy at least for partial epilepsy recruited in current study, similar analyses were conducted in MPE. The spatial maps of the eight RSNs for MPE are illustrated in Figure 1. It can be seen that the spatial pattern of the RSNs in MPE is similar to that identified in HC and TLE. The results of the group comparisons of functional connectivity between controls and MPE are shown in Figure 3. We found that MPE presented almost the same trend as TLE in functional connectivity alterations. Specifically, all the networks revealed decreased functional connectivity within the regions of each network. Among the eight RSNs, the right lateral DAN (RSN 6) and the visual network(RSN 7) revealed both reduced and increased functional connectivity. The anatomical coordinates of significant clusters, including their t value magnitudes and the Brodmann areas in which activations occurred are reported in Table 2. Furthermore, the comparison between two patient groups was performed. No significant difference in RSN2 and RSN7 was found between TLE and MPE. The significant increased functional connectivity was observed in RSN3, and decreased functional connectivity was found in RSN1, RSN4, RSN5, RSN6, and RSN8 in MPE compared to TLE. The maps of difference were revealed in the Figure 4. Table 3 summarized the significantly difference functional connectivity in each RSN.

**Table 2:** Significant difference of functional connectivity of the eight RSNs between HC and MPEG.

RSNs	Patients with MPE vs Controls			
	Brain regions	Brodmann	Peak locationMNI (x , y, z)	Peak t value
RSN1	Left Posterior Cingulate	23	−6, −44,27	15.21
	Right Posterior Cingulate	23	7, −48,30	11.05
	Left Precuneus Gyrus	23	−4, −62,28	10.50
	Right Precuneus Gyrus	31	5, −64,29	6.23
	Left Angular	39	−46, −66,29	4.56
RSN2	Left Superior Frontal Gyrus,	8	0,48,48	13.28
	Left Anterior Cingulate	24	−5,23,29	8.03
	Right Anterior Cingulate	24,33	9,15,21	5.70
RSN3	Left Supplementary Motor Area	6	−1, −15,53	7.60
	Right Cingulate Gyrus	24,32	3,6,42	5.79
	Left Cingulate Gyrus	24	−5,3,35	5.43
	Left Postcentral Gyrus	3	−31, −36,55	4.72
RSN4	Left Anterior Cingulate	32	−3,33, −3	14.77
	Left Medial Frontal Gyrus	11	−12,24, −9	11.72
	Right Medial Frontal Gyrus	11	5,48, −12	8.80
RSN5	Left Inferior Parietal Lobule	39	−45, −50,39	10.73
	Left Inferior Frontal Gyrus	45	−41,43,16	8.98
	Left Middle Temporal Gyrus	37	−42, −57,24	5.74
RSN6	Right Inferior Parietal Lobule	40	54, −54,39	9.76
	Right Angular	39	56, −54,34	8.38
	Right Anterior Cingulate	10,32	15,42,0	7.65
	Right Superior Frontal Gyrus	8	9,42,45	7.00
	Right Anterior Cingulate	32	18,27, −9	5.90
	Right Inferior Frontal Gyrus*	44	49,15,34	8.94
RSN7	Left Superior Occipital Gyrus	17	−8, −98,10	10.98
	Right Superior Occipital Gyrus	17	21, −99,7	9.95
	Left middle Occipital Gyrus*	18	−31, −89,7	12.31
	Right middle Occipital Gyrus*	19	39, −77,10	11.86
	Right Lingual Gyrus*	17	8, −64,11	9.60
	Left Cuneus*	18	−12, −95,9	8.92
	Right Cuneus*	17	9, −95,5	6.90
RSN8	Left Insula	13	−42,9, −3	12.93
	Left Parahippocampal Gyrus	28,34	−24, −9, −12	8.99
	Right Insula	13	36,12, −6	6.61
	Right Superior Temporal Gyrus	22	51, −5,0	4.45

Note: * regions represented increased functional connectivity in MPE in contrast to HC.

**Table 3:** Significant difference of functional connectivity of the eight RSNs between MPEG and TLEG.

RSNs	Patients with MPE vs Patients with TLE			
	Brain regions	Brodmann	Peak locationMNI (x , y, z)	Peak t value
RSN1	Right Posterior Cingulate	23	5, −41,29	7.51
RSN3	Left Paracentral Lobule*	4	−12, −27,81	10.61
	Left Postcentral Gyrus*	3	−35, −33,51	9.73
	Left Precentral Gyrus*	4	−29, −28,57	9.00
	Right Postcentral Gyrus*	3	37, −36,60	7.03
RSN4	Right Anterior Cingulate	11	5,34, −3	10.51
	Right Medial Frontal Gyrus	10	3,54, −3	7.09
	Right Superior Frontal Gyrus	11	21,54,12	4.26
RSN5	Left Inferior Parietal Lobule	40	−54, −60,48	8.46
	Right Inferior Parietal Lobule	40	55, −57,42	8.23
	Left Superior Frontal Gyrus	6	−9,39,60	5.51
RSN6	Right Inferior Parietal Lobule	40	45, −54,45	9.29
	Right Angular Gyrus	39	43, −62,41	7.72
	Right Middler Frontal Gyrus	9	33,38,40	7.11
	Right Superior Frontal Gyrus	9	8,46,43	4.59
RSN8	Left Insula	13	−48, −39,18	12.25
	Left Middle Temporal Gyrus	21	−57, −48,9	10.12
	Right Postcentral Gyrus	35,7	24,−45,66	5.34
	Left Superior Temporal Gyrus	22,42	−55, −35,9	10.00
	Right Inferior Parietal Lobule	40	39,−45,57	5.42
	Right Middle Temporal Gyrus	21,22	60,−48,3	5.49

Note: * regions represented increased functional connectivity in MPE in contrast to TLE.

### FNC analysis between groups

**Figure 5:**
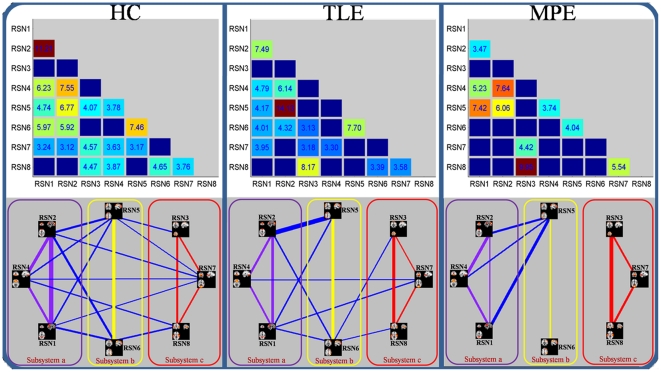
Correlation matrices representing results of FNC analysis for HC (right), TLE (middle) and MPE (left), Significant connections (P<0.05 FDR-corrected) were marked by corresponding T values at upper of figure. The network map was showed at bottom of figure. Three subsystems: a) these RSNs (in pink rectangle) related to information integration and modulation including the posterior part of the DMN (RSN1), anterior part of the DMN (RSN2) and self-referential network (RSN4); b) these RSNs (in yellow rectangle) related to higher level cognition including the DAN (RSN5,RSN6); c) these RSNs(in red rectangle) related to primary function including the sensorimotor network (RSN3), visual network (RSN7) and auditory network (RSN8). The intensity of the temporal dependency between RSNs was indicated by the thickness of the corresponding line.

Using one-sample t-test, 19 out of the 28 possible combinations was significant in HC group, 15 in TLE group and 10 in MPE group. The results were shown in Figure 5, and some disconnections were found in two patients groups. In order to further understand the architecture of the FNC, a network reorganization procedure was conducted, Eight RSNs were divided into 3 subsystems: a) the RSNs related to information integration and modulation including the posterior part of the DMN (RSN1), anterior part of the DMN (RSN2) and self-referential network (RSN4); b) the RSNs related to higher level cognition including the DAN (RSN5,RSN6); c) the RSNs related to primary function including the sensorimotor network (RSN3), visual network (RSN7) and auditory network (RSN8); The reorganization results of the FNC for HC, TLE and MPE are shown in bottom of Figure 5. It was interesting that compared with controls the intra-system connections were preserved for all the three subsystems in two patients groups, the lost connections were confined to intersystem connections. This finding may indicate that the FNC impairment in patients with epilepsy has a hierarchical selectivity.

To understand the difference of each FNC between groups, two-sample t-tests was performed on all 28 possible combinations. However, no significant difference (P<0.05, FDR-corrected) was found in each combination between groups (between HC and two patients’ groups, between two patients’ groups respectively). It is seemingly ambivalent with the dis-connectivity in two patients’ groups aforementioned. One explanation for this phenomenon is that the selective impairment of FNC aforementioned focused on the system level, but the finding, no significant difference in each combination, was inclined to explore the differences of interaction in two RSNs between groups. Besides, the small sample size may be anther reason to worsen statistic significance.

## Discussion

Low-frequency BOLD fluctuation was reportedly altered in many neurological and psychiatric disorders such as Alzheimer’s disease [23], [41], [42], epilepsy [30], [43] and schizophrenia [44], [45]. In current study, aiming to investigate the functional connectivity inter- and intra-RSNs in patients with partial epileptic seizures, eight RSNs were select to conduct a systematical resting-state network analysis in a cohort of partial epilepsy patients and healthy controls. Compared with the controls, two remarkable findings were found in dataset of patients with TLE and MPE. First, decreased functional connectivity was found within all the RSNs extensively. Second, the networks constructed by FNC analysis were disconnected. Interesting, by dividing the 8 RSNs into 3 subsystems we found that intra-system connections were preserved for all the three subsystems while the lost connections were confined to intersystem connections.

### Alteration of functional connectivity

Recently, the functional connectivity analysis focused on RSNs may implicated some underlying or intrinsic interaction among the discrete neuroanatomical regions [18], [46], and studying brain in the perspective of resting-state network may contribute to the understanding of neuropathophysiological mechanisms in some brain disorders. In current study, decreased functional connectivity was found in all eight RSNs in two groups of patients. In previous studies focused on TLE, decreased functional connectivity was found in DAN, DMN and perception networks. Here, the consistent results were found in TLE, and similar results were observed in patient with MPE. These results suggested that the decreased resting state functional connectivity might be a remarkable characteristic of partial epilepsy. The changes on each RSN might implicated the various functional abnormality responded to the RSN.

Self-referential network is a network including mainly the medial-ventral prefrontal cortex and the pregenual anterior cingulate, putatively related to self-referential mental activity [38]. In current study, RSN4 was the network overlapping with the self-referential network, and decreased function connectivity in this RSN was found in TLE and MPE. In a recent simultaneous EEG-fMRI study, using independent component analysis on the fMRI data, the self-referential network was identified, and was strongly associated with the EEG power spectra of gamma rhythm [47]. During spike-wave activity, gamma synchronization is significantly decreased [48], and the decreased synchronization may result in functional abnormality in self-referential network in TLE and MPE. Besides, the altered functional architectural integration in self-referential network may reflect the impairment of brain function related to self-referential processing during seizure, such as, absence of self-awareness, emotional and psychic experiences in TLE [49]. Further investigations based on simultaneous EEG and fMRI [50] may reveal the details about the impairment of self-referential network. The other RSN, DMN, was associated with the tasks about oneself involving autobiographical memory or future prospection [20], [51]. It is a common observation in brain imaging research that a specific set of brain regions is engaged when individuals are not focused on the external environment. One possible function of DMN is that it may play an important role in constructing dynamic mental simulations based on past experiences, e.g. those used during remembering. Another function of DMN is to support exploratory monitoring of the external environment when focused attention is relaxed [51]. Abnormal activities of the default-mode brain network have been reported in various brain disorders, such as Alzheimer’s disease [41], [42], [52] , autism [53], schizophrenia [45], epilepsy [30], [43]. In our previous study, we have observed the altered functional connectivity in DMN in absence epilepsy. Here, Consistent with the precious studies in TLE [26]–[28], the decreased functional connectivity in DMN was found in TLE and MPE. These findings indicated DMN abnormalities in patients with partial epilepsy.

Among those already known RSNs, dorsal attention network (DAN) is the other RSN investigated in healthy or mental disorder widely. The functional connectivity disturbance of DAN may be related with the behavior abnormality in Attention-Deficit/Hyperactivity Disorder (ADHD) [54], autism [55] and epilepsy [29]. In line with previous study [29], decreased functional connectivity was found in DAN in TLE group and MPE group. We infer that this decreased functional connectivity may explain the attention deficit which is a common symptom in the epilepsy patients. There is interesting founding that the increased functional connectivity was found at the right supper frontal lobule in MPE compared with the controls (RSN6). Four patients with the benign childhood epilepsy with centrotemporal spikes (BECT) were included in the MPE group. The regions with increased functional connectivity may related to the source of centrotemporal spikes [56]. Although the intracerebral EEG recording also suggested that the network with a reinforced functional connectivity may be a feature of epileptogenic cortex [57], [58], we will recruit more patients with BECT to validate the presumption.

The networks related to perception have been studied in TLE [25]. In current study, three RSNs (auditory, motor and visual network) representing the perceptual networks were selected. Decreased functional connectivity was found in auditory network, visual network and somatosensory network both in two patients group and the results were similar with previous study [25]. Among the eight RSNs, visual network was the only network that displayed increased functional connectivity. Zhang, et al. have mentioned this phenomenon in a previous fMRI study of TLE [25]. The results indicated that auditory and motor function might be affected by epilepsy, but the primary visual function may not be reduced.

As mentioned above, TLE and MPE revealed almost the similar trend on the functional connectivity alteration in all of the eight RSNs (the regions observed altered functional connectivity alteration in TLE and MPE were mostly overlapped). The results might suggest that decreased resting state functional connectivity was a remarkable characteristic of partial epilepsy. This decreased resting state functional connectivity might provide evidence for the functional impairment in partial epilepsy patients in the perspective of resting-state fMRI. However, there were some subtle differences between the functional connectivity alteration of TLE and MPE. Furthermore, the two-sample t-test was processed between the MPE and TLE. The results in MPE compared to TLE revealed that the significantly decreased functional connectivity was found in RSN1, RSN4, RSN5, RSN6 and RSN8. Four types of partial epilepsy were included in MPE. We presumed that the inconsistency of epilepsy types might caused the reduced statistic eigenvalue in several RSNs with decreased functional connectivity in MPE in contrast to TLE. Besides, increased functional connectivity was only observed in RSN3 in MPE compared with TLE, it may implicate that the motor abnormality was more serious in patients with TLE than patients with MPE include in current study. It was consistent with the result between patients and controls, which the extent of voxels involved decreased functional connectivity in the TLE was larger than that in the MPE (figure 2, and figure 3).

### Dis-connectivity of FNC

Significant temporal dependency may exist between the RSNs resulted by spatial ICA. As an extension of functional connectivity, FNC has been increasingly used as an effective method to evaluate the information interaction between RSNs in healthy population [59] and patients with various brain disorders [33], [60], [61] in fMRI studies. To our knowledge, this is the first study that focused on the FNC by used resting-state fMRI in epilepsy. In current study, a constrained maximal time-lagged correlation was computed for all of the network combinations. Dense FNCs observed among the networks constructed by HC, and that may indicate the strong information communication between RSNs in healthy controls. On the contrary, widespread disconnections of FNC were found in the TLE as well as MPE (Figure 5). The dis-connectivity may relate to the functional impairment imposed by partial epilepsy. Furthermore, the disconnection was more in MPE than TLE. As mentioned above, we presumed that this strong disconnection in MPE might result from the inhomogeneity of this dataset in current study. Different types of partial epilepsy might impose influence on different brain regions, resulting in corresponding alteration of connections between RSNs. Different brain connection impairment in different patients led to the insignificancy of most of the connections. Besides, the left and right TLE were included in TLE group, that may also cause inhomogeneous, because an effect of epilepsy lateralization on connectivity alteration has been demonstrated in TLE [62]. Here, we thought that the inference for the result in current study may be slight. The reasons included that the RSNs selected in current study were not directly correlated to the epileptogenic zeros, and they were symmetric in brain except RSN5 and RSN6 (but symmetry between them). However, the deficiency should be considered in future.

Additionally, conducting a reorganization procedure by dividing the eight RSNs into three subsystems, we found that the intra-system connections were preserved for all the there subsystems in two groups of patients with partial epilepsy, while the lost connections were confined to intersystem. The partition has constructed a hierarchical structure with two levels in FNC. The intersystem connections were in the high level of the hierarchy while intra-system connections were in the low level of the hierarchy. Obviously, the patients with partial epilepsy preferred to impair the intersystem connections in highest level of the hierarchy but not the intra-system connections. The lose interaction among the intersystem might be associated with the disturbance of the high level complex function which needed the integrated multi-systems, such as learning [63], memory and language processing [64]. The remained interaction among the intra-system might be a basic condition to support the normal behavior and function in interictal period. This phenomenon might indicate that the FNC impairment in patients with epilepsy had a hierarchical selectivity, and the selective impairment had an important functional and theoretical implication that it was unsuitable to understand the partial epilepsy only from global or local perspective.

### Methodological considerations and limitations

However, there do exist some uncontrolled methodological confounds that may affect our results. First, there are some pitfalls about ICA to be mentioned hereby. How to choose the number of independent components and how to threshold the IC maps are still open questions. In this work, the minimum description length criterion implanted in GIFT was used to determine the number of ICs. On the other hand, the employment of physiological information is hard for current algorithm framework of ICA. Hybrid approach based on ICA and Bayesian may be an acceptable amelioration[50]. Second, the anti-epileptic drug (AED) may have influence on functional connectivity despite the fact that the patients had discontinued medication for about 24 h in the present study. A recent work has shown that midazolam tends to increase functional connectivity parameters in primary sensory and sensorymotor neural networks [65]. Third, the study involved a small sample size—just 16 partial epilepsy patients, and the age of these patients was in a wide range (8∼35years). Due to the limited sample size, only five types of partial epilepsy were recruited, future studies should include larger sample sizes to determine these mechanisms found in current study. At last, Similar as these patients, healthy controls with a wide age range (9∼30 years old) were selected. The potential intra-group heterogeneity was leaded in two groups. Pearson’s correlation analysis was processed between each FNC and the age of subjects for each group respectively. No significant correlation was found in each group (P<0.05, FDR-corrected). Though, the negative result was observed, we need to take care of the influence of the age difference. The potential solution included that large size samples are recruited, and the age was use as covariance in the comparison between groups.

In summary, we examined the influence that partial epilepsy exerted on the RSNs systematically in the present study. Compared with the controls, decreased functional connectivity within all RSNs was found in both TLE and MPE. However, in contrast to TLE, we found decreased functional connectivity in 5 RSNs increased functional connectivity in 1 RSN, and no difference in the other 2 RSNs in MPE. Furthermore, the hierarchical disconnections of FNC was found in two patient groups, in which the intra-system connections were preserved for all three subsystems while the lost connections were confined to intersystem connections in patients with partial epilepsy. These findings might suggest that decreased resting state functional connectivity and disconnection of FNC are two remarkable characteristics of partial epilepsy. The selective impairment of FNC had an important functional and theoretical implication that it was unsuitable to understand the partial epilepsy only from global or local perspective. We presumed that studying epilepsy in the multi-perspective based on RSNs might be a valuable means to assess the functional changes corresponding to specific RSN, and contribute to our understanding of the neuro-pathophysiological mechanism of epilepsy.

## Materials and Methods

### Participants

A total of 16 right-handed patients with partial epilepsy were recruited from West China Hospital of Sichuan University, Chengdu, China. The clinical patient details are shown in Table 4. The seizure subtypes were based on the International Classification of the Epilepsies (Commission on Classification and Terminology of the International League against Epilepsy, 1981), and the recruitment was made based on video evidence, EEG telemetry, scalp EEG, and clinical manifestations. All patients were seizure-free for at least one day and discontinued medication for about 24 h prior to MRI scanning. A total of 14 gender- and age-matched right-handed controls were also recruited (9∼30years old). None of the controls had neurological or psychiatric disorders. The study was approved by the Ethics committee of the West China Hospital, and was performed according to the standards set by the Declaration of Helsinki. Written informed consent was obtained from each participant or parents (for children).

**Table 4:** Clinical details of Patients.

Case	Gender	Age(Year)	SeizureType	MRI	Epilepsy syndrome	Anti-epileptic drug	
1	M	15	CPS	L Hippocampal Sclerosis	mTLE	Carbamazepine	
2	M	16	CPS	L Hippocampal Sclerosis	mTLE	Topiramate	
3	F	15	CPS	L Hippocampal Sclerosis	mTLE	Carbamazepine	
4	M	17	CPS	L Hippocampal Sclerosis	mTLE	Carbamazepine	
5	F	30	CPS	R Hippocampal Sclerosis	mTLE	None	
6	F	22	CPS	L Hippocampal Sclerosis	mTLE	None	
7	M	10	CPS	R Hippocampal Sclerosis	mTLE	None	
8	F	11	CPS	Normal	BECT	None	
9	F	14	CPS	R occipital lobeGray matter heterotopia	OLE	None	
10	F	8	CPS	Normal	BECT	Oxcarbazepine	
11	M	35	SPS	R frontal-parietal lobe lesion	FPLE	None	
12	M	25	CPS	L occipital lobeGray matter heterotopia	OLE	None	
13	M	14	CPS	L frontal lobeFCD	FLE	None	
14	M	19	SPS	Normal	FLE	None	
15	F	9	CPS	Normal	BECT	valproic acid	
16	M	11	CPS	Normal	BECT	None	

SPS/CPS: Simple/Complex Partial Seizure; FCD: Focal Cortical Dysplasia; BECT: Benign Epilepsy of Childhood with Central-temporal spikes; mTLE: mesial temporal lobe epilepsy; OLE: occipital lobe epilepsy; FLE: frontal lobe epilepsy; R/L: right/left.

### MRI data acquisition

MRI data were acquired on a 3T MRI system (EXCITE, GE Milwaukee, USA) with an eight-channel phased array head coil in Huaxi MR Research Center (HMRRC), Department of Radiology, West China Hospital of Sichuan University, Chengdu, China. Structural T1-weighted images were acquired in axial orientation using a three-dimensional (3D) spoiled gradient recalled (SPGR) sequence (TR = 8.5 ms, TE = 3.4 ms, FOV = 24 cm×24 cm, flip angle = 12°, matrix = 512×512, 156 slices) with a voxel size of 0.96×0.96×1.00 mm^3^. MR images sensitized to changes in BOLD signal levels (TR = 2000 ms, TE = 30 ms, FOV = 24 cm×24 cm, flip angle = 90°, matrix = 64×64, 30 slices) were obtained by a gradient-echo echo-planar imaging (EPI) sequence. The slice thickness was 5 mm (no slice gap) resulting in a voxel size 3.75×3.75×5.00 mm^3^. According to patient endurance, 2∼3 resting-state fMRI runs were performed, and each functional run contained 205 image volumes. The first five volumes were discarded to ensure steady-state longitudinal magnetization. During the resting-state scan, participants were instructed simply to keep their eyes closed and not to think of anything in particular. The fMRI data of 30 participants were further divided into 3 groups: the healthy controls (HC) included 14 healthy controls, TLE group included 7 patients with temporal lobe epilepsy, and MPE group contained 9 patients with other type of partial epilepsy patents (see Table 4).

### Data preprocessing

Before submitted to ICA, fMRI data were preprocessed using the Statistical Parametric Mapping software package, SPM2 (http://www.fil.ion.ucl.ac.uk/spm/). For each subject, all EPI images were first corrected for the temporal difference and head motion correction. Realigned images were spatially normalized to the Montreal Neurological Institute (MNI) EPI template in SPM2, then each voxel was resampled to 3×3×3 mm^3^. Finally, all images were spatially smoothed using an isotropic Gaussian filter (8 mm full width half-maximum [FWHM]). The translation and rotation were checked, and the images with head movement greater than 2 mm in any direction or head rotation greater than one degree were excluded.

### Identification of the RSNs and analysis within RSNs

Group spatial ICA was used to decompose all the data into independent components using the GIFT software (http://icatb.sourceforge.net/) [66]. GIFT contains three distinct stages: (1) data reduction, (2) application of the ICA algorithm, and (3) back reconstruction. The two-stage PCA was performed in data reduction step to avoid the result variability cause by the three-stage reduction [67]. To determine the number of independent components (ICs), dimension estimation on all subjects was performed using the minimum description length (MDL) criterion [68]. Subsequently, the infomax algorithm [69] was used in independent component estimation. In the back reconstruction step, the dual-regression (DR) approach, which has been shown to be a test-retest reliable method to explore ICs [67], was used to back-reconstruct the individual subject components. The IC time-courses and spatial maps for each participant were acquired for following processing, and the subject-specific maps were converted to z score.

Both the spatial pattern and frequency spectra of each component were visually inspected to determine their appearance as potential RSNs or possible image artifacts. The IC time-courses were transformed into frequency domain. If the proportion of powers in >0.1 Hz was more than 50% of the total power, this component would be discarded because the potential RSNs would show dominant power in the expected ‘very low frequency’ domain. At last, eight components were selected as of interest for further analyses. For each of the eight RSNs, z-maps in each group were then gathered for a random-effect analysis using the one-sample t-test in SPM2 respectively. The thresholds was set at P<0.05 with a false discovery rate (FDR) criterion. Subsequently, to investigate the functional connectivity changes in each RSN, the z-maps of the RSN were compared between groups using two-sample t-tests (P<0.05, corrected by FDR). Particularly, in each RSN, we restricted the two-sample t-tests to only including the voxels within a mask, which defined by the one-sample t-test of IC result of the subjects in the control group.

### FNC analysis between RSNs

The ICA algorithm assumes that the time courses of brain regions within one component are synchronous [70]. Though the components resulted by spatially ICA have optimized independence in spatial domain, the spares of components in brain fMRI is also considered as anther important feature in ICA algorithm, such as infomax algorithm [71]. The significant temporal dependency may exist between components. In order to examine the possible interaction between the RSNs, a constrained maximal time-lagged correlation method was adopted [33]. The time courses of components for all subjects were first interpolated to enlable detection of sub TR hemodynamic delay difference. Subsequently, the time courses were filtered through a band-pass filter, with frequencies between 0.01 Hz and 0.1 Hz. In the present study, the time lag circularly shifted from−5 to +5 s resulting in 11 correlation coefficients for one combination of each subject. As 8 components were identified, the number of pair-wise combinations is 28 for each subject. The maximal lagged correlation was then picked up from the 28 combinations. At last, the temporal interaction between any 2 RSNs of the 8 total components were examined by the one-sample t-test (P<0.05, corrected by FDR). Statistically significant correlation combinations from the 28 possible combinations were extracted for patients and controls, resulting in maps of FNC for each group separately. To understand the difference of each FNC between groups, two-sample t-tests were performed on all 28 possible combinations, and the statistical significance level was set to P<0.05 (FDR-corrected).
